# Prostaglandin E-major urinary metabolite versus fecal immunochemical occult blood test as a biomarker for patient with ulcerative colitis

**DOI:** 10.1186/s12876-020-01256-5

**Published:** 2020-04-19

**Authors:** Natsuki Ishida, Takahiro Miyazu, Ryosuke Takano, Satoshi Tamura, Shinya Tani, Takuma Kagami, Mihoko Yamade, Yasushi Hamaya, Moriya Iwaizumi, Satoshi Osawa, Takahisa Furuta, Hiroaki Miyajima, Ken Sugimoto

**Affiliations:** 1 First Department of Medicine, Hamamatsu University School of Medicine, 1-20-1 Handayama, Higashi-ku, Hamamatsu, 431-3192 Japan; 2 Department of Endoscopic and Photodynamic Medicine, Hamamatsu University School of Medicine, Hamamatsu, Shizuoka, Japan; 3 Department of Laboratory Medicine, Hamamatsu University School of Medicine, Hamamatsu, Shizuoka, Japan; 4 Center for Clinical Research, Hamamatsu University School of Medicine, Hamamatsu, Shizuoka, Japan

**Keywords:** Ulcerative colitis, Inflammatory bowel disease, Prostaglandin E-major urinary metabolite, Fecal immunochemical occult blood test

## Abstract

**Background:**

Prostaglandin E-major urinary metabolite (PGE-MUM) may be a novel biomarker for evaluating disease activity in ulcerative colitis (UC). We compared its usefulness to that of the fecal immunochemical occult blood test (FIT).

**Methods:**

PGE-MUM and FIT measurements were performed of 92 urinary and fecal samples obtained from 60 patients with UC. Endoscopic activity was determined by Mayo endoscopic subscore (eMayo) and Ulcerative Colitis Endoscopic Index of Severity (UCEIS) score.

**Results:**

PGE-MUM levels and FIT results showed a significant correlation with respect to eMayo (*P* <  0.001 and *P* < 0.001, respectively), and there was a significant difference in PGE-MUM values between the groups below eMayo1 and above eMayo2 (*P* = 0.012). Both biomarkers were significantly correlated with the UCEIS score (*P* < 0.001 and *P* < 0.001, respectively), and the PGE-MUM values were significantly different between groups below UCEIS1 and above UCEIS2 (*P* = 0.012). PGE-MUM and FIT were significantly correlated with eMayo in the group with a disease duration < 5 years (*P* = 0.041 and *P* < 0.001, respectively). Although PGE-MUM and eMayo differed significantly between groups over 5 years (*P* = 0.012), FIT was not correlated with eMayo (*P* = 0.101).

**Conclusions:**

PGE-MUM is useful as a biomarker as FIT for evaluating the endoscopic activity, particularly in long-term affected patients with UC.

## Background

Ulcerative colitis (UC) is an idiopathic chronic inflammatory disorder characterized by diarrhea, bloody stool, abdominal pain, fever, anemia, and weight loss [1], and its clinical course is characteristic of repeating remission and relapse [2]. Although assessing the disease activity and response to treatment is important to achieving better disease control, current studies have recommended that achieving mucosal healing is the treatment goal of UC since it is associated with sustained clinical remission, decreased hospitalization rates, and the avoidance of colectomy [3]. Endoscopic evaluations are important because patients with UC can be asymptomatic despite the presence of mucosal inflammation. However, colonoscopy is expensive and invasive, making frequent examinations difficult. Therefore, non-invasive biomarkers of mucosal status with accuracy comparable to that of endoscopy are needed.

Serum C-reactive protein (CRP) is the most common biomarker used to assess inflammation in patients with IBD. However, some reports have stated that CRP levels may remain within the physiological range or only show mild abnormalities even in patients with endoscopically active UC [4]. Fecal calprotectin (FC) and the fecal immunochemical occult blood test (FIT) are minimally invasive techniques that reflect the endoscopic severity of UC [5–8]. However, fecal samples can be more difficult to obtain than blood or urine samples because patients are reluctant to bring fecal samples to the hospital and it is difficult to collect fecal sample from diarrhea stool. However, these problems can be circumvented by examinations using urine samples. Arai et al. reported that prostaglandin E-major urinary metabolite (PGE-MUM) was a reliable biomarker of the colonoscopic and histological appearance of UC, suggesting that it was more sensitive than those previously utilized to evaluate UC-related mucosal inflammation [9]. Here we investigated the usefulness of PGE-MUM versus FIT as a biomarker for evaluating the endoscopic activity of patients with UC.

## Methods

### Patients and study design

Sixty patients with UC treated at Hamamatsu University School of Medicine between August 2016 and August 2018 were included in this study. Colonoscopy was performed a total of 92 times for these 60 patients. In this study, almost all the patients received laxative administration on the day before colonoscopy. Each time, urine and fecal specimens were collected; PGE-MUM and FIT were measured; and endoscopic severity, clinical activity, and blood test results were prospectively evaluated. The diagnosis of UC was based on typical medical history and clinical features on endoscopic and histological assessments according to current guidelines. UC is a chronic inflammatory condition with a relapsing and remitting course. Patients who were not diagnosed with UC, such as those with indeterminate colitis or unclassified inflammatory bowel disease, were excluded. Smokers were also excluded since smoking can reportedly increase PGE-MUM values [10]. Since the complication of chronic fibrosing interstitial pneumonia increases PGE-MUM values, patients with interstitial pneumonia were also excluded [11].

### Disease assessment

Clinical disease activity was evaluated using Lichtiger’s clinical activity index (CAI), which is based on the following criteria: diarrhea (number of daily stools), nocturnal diarrhea, visible blood in stool (percentage of movements), fecal incontinence, abdominal pain or cramping, general well-being, abdominal tenderness, and need for anti-diarrheal drugs [12]. Total scores range from 0 to 21 points. CAI was evaluated on the same day as the endoscopic examination, and clinical remission was defined as a CAI ≤ 4.

Patients underwent colonoscopy after performing bowel preparation consisting of a polyethylene glycol–based electrolyte solution or glycerin enema. UC mucosal status was assessed using the Mayo endoscopic subscore (eMayo) classification system and the Ulcerative Colitis Endoscopic Index of Severity (UCEIS) score [13, 14]. The eMayo scores were as follows: 0, normal or inactive disease; 1, mild disease with erythema, decreased vascular pattern, mild friability; 2, moderate disease with marked erythema, absence of vascular patterns, friability, erosions; and 3, severe disease with spontaneous bleeding, ulceration. The UCEIS scores were calculated as the simple sum of three descriptors: vascular pattern (score 0–2), bleeding (score 0–3), and erosions and ulcers (score 0–3). These were evaluated at the most active lesion within the colon. Endoscopic remission and mucosal healing were defined as eMayo scores of 0 or 1 and UCEIS scores of 0 or 1. As PGE-MUM reflects the inflammation status of the whole colon, we investigated the correlation of PGE-MUM or FIT to the sum of the eMayo calculated from six segments (the cecum, ascending colon, transverse colon, descending colon, sigmoid colon, and rectum).

### PGE-MUM analysis

Urine samples were obtained on the morning of the endoscopic examination at our hospital and sent to the SRL Hachioji Laboratory (Tokyo, Japan). Each spot urine sample was measured by a ɤ-counter (Hitachi) using a Bicylic PGE-MUM RIA Kit (Fuji Rebio, Tokyo, Japan). The measured PGE-MUM values were corrected with urinary creatinine.

### Fecal immunochemical test analysis

Fecal samples were prepared on the day before or of the colonoscopy to prevent bleeding due to the endoscopic examination. Patients collected a stool specimen by using a collection kit (Eiken Chemical, Tokyo, Japan). The submitted samples were immediately processed and examined using OC Sensor io (Eiken Chemical). The FIT results were obtained on the day of or within 1 week of colonoscopy.

### Statistical analysis

Patient demographics and baseline characteristics were summarized using descriptive statistics. Correlations between two biomarkers (PGE-MUM and FIT) and other values or activity index values were analyzed with logistic regression analysis. Intergroup differences were compared using Student’s t test. Statistical analyses of the data were performed using the Excel statistical software package (Ekuseru-Toukei 2010; Social Survey Research Information Co., Ltd., Tokyo, Japan) and the statistical program R (http://cran.r-project.org).

### Ethical statement

Informed consent was obtained from all patients after explanation of the purpose of the study and the nature of the procedures involved. The study protocol was reviewed and approved by the Institutional Review Board of Hamamatsu University School of Medicine (number 18–228). Further, the investigation was conducted in accordance with Good Clinical Practice principles in adherence to the Declaration of Helsinki.

## Results

### Patient characteristics

The patients’ baseline characteristics are shown in Table 1. Urinary and fecal samples (92 specimens each) were collected from the 60 patients with UC. The mean patient age was 48.5 years (Table 1). Mean disease duration was 7.2 years (range, 0.1–26). With regard to disease extent, 57 (63.0%) patients had extensive colitis, 24 (26.1%) had left colitis, and 11 (10.9%) had proctitis. Regarding the treatment received at the time of sampling, 58 (63.0%) patients were taking oral 5-aminosalicylic acid (5-ASA), 12 (13.0%) were taking suppository 5-ASA, 12 (13.0%) were taking systematic steroids, 35 (38.0%) were taking immunomodulators, 10 (10.9%) were taking tacrolimus, and 25 (27.2%) were taking anti-tumor necrosis factor-alpha therapy. The mean PGE-MUM and FIT levels were 29.2 μg/g∙Cr and 2178 ng/mL, respectively.

**Table 1:** Demographic features of a total of 92 patients with UC

Characteristics	***N***=92
Age(year), mean(range)±SD	48.5 (14-83) ±16.3
Male/Female, n(%)	64/28 (69.6/30.4)
Disease duration(year), mean(range)±SD	7.2 (0.1-26) ± 7.1
Disease extent, n(%)	
Extensive colitis	57 (63.0)
Left sided colitis	24 (26.1)
Proctitis	11 (10.9)
CAI(Lichtiger socre) mean(range)±SD	3.4 ± 4.3
eMayo mean(range)±SD	1.0 (0-3) ±1.0
eMayo 0, n (%)	38 (41.3)
eMayo 1, n (%)	21 (22.8)
eMayo 2, n (%)	27 (29.3)
eMayo 3, n (%)	6 (6.6)
UCEIS mean(range)±SD	2.1 (0-7) ± 1.9
PGE-MUM (μ·g^-1^·Cr^-1^) mean(range)±SD	29.2 (5.1-93.9) ± 18.5
Fecal hemoglobin concentrations (ng/ml), n (%) mean(range)±SD	2178 (0-26500) ± 4667
Drug at study, n (%)	
Oral 5-ASA	58 (63.0)
Suppository 5-ASA	12 (13.0)
Systemic steroids	12 (13.0)
Azathioprine / Mercaptopurine	35 (38.0)
Tacrolimus	10 (10.9)
Biologics	25 (27.2)

*CAI* clinical activity index, *eMayo* Mayo endoscopic subscore, *UCEIS* Ulcerative Colitis Endoscopic Index of severity, *PGE-MUM* Prostaglandin E-Major Urinary Metabolite, *5-ASA* 5-aminosalicylic acid

### Correlation between urinary/fecal biomarkers and blood biomarkers in patients with UC

**Fig. 1:**
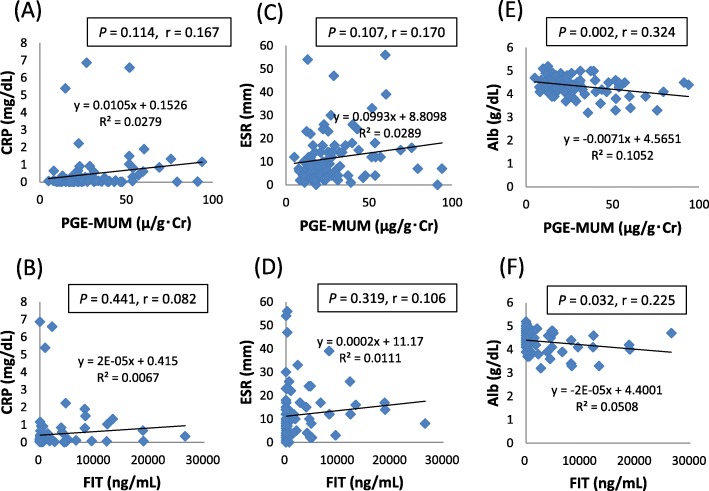
Correlation between urinary/fecal biomarkers and blood biomarkers in patients with ulcerative colitis (UC). Scatter plots of (**a**) prostaglandin E-major urinary metabolite (PGE-MUM) vs. C-reactive protein (CRP), (**b**) fecal immunochemical occult blood test (FIT) vs. CRP, (**c**) PGE-MUM vs. erythrocyte sedimentation rate (ESR), (**d**) FIT vs. ESR, (**e**) PGE-MUM vs. serum concentration (Alb), and (**f**) FIT vs. Alb

We next investigated the correlation between PGE-MUM and the blood biomarkers CRP and erythrocyte sedimentation rate (ESR), which represented the inflammatory condition of UC, and the blood biomarker serum albumin (Alb), which represented nutritional condition. Although PGE-MUM showed no significant correlation with CRP and ESR, PGE-MUM showed a significant negative correlation with Alb (*P* = 0.002) (Fig. 1a-c). We also investigated whether FIT was significantly correlated with CRP, ESR, and Alb. Although FIT showed no significant correlation with CRP or ESR, there was a significant negative correlation between FIT and Alb (*P* = 0.032) (Fig. 1d-f).

### Correlation between urinary/fecal biomarkers and endoscopic activity index in patients with UC

**Fig. 2:**
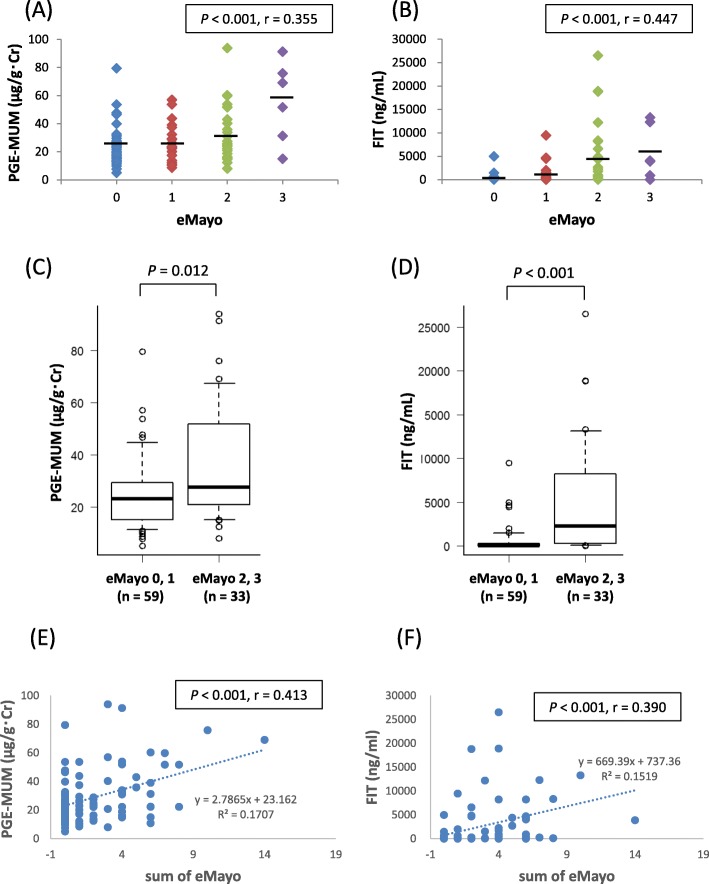
Correlation between urinary/fecal biomarkers and Mayo endoscopic subscore (eMayo) in patients with ulcerative colitis (UC). Scatter plots of (**a**) eMayo vs prostaglandin E-major urinary metabolite (PGE-MUM), and (**b**) eMayo vs fecal immunochemical occult blood test (FIT). **c** Comparison of PGE-MUM level between subgroups (eMayo ≤1 and eMayo ≥2). **d** Comparison of FIT level between groups with eMayo ≤1 and eMayo ≥2. Scatter plots between (**e**) the sum of eMayo and the prostaglandin E-major urinary metabolite (PGE-MUM), and (**f**) the sum of eMayo and the fecal immunochemical occult blood test (FIT)

All patients in this study underwent colonoscopy as well as evaluations of endoscopic severity using eMayo and UCEIS. We initially examined the correlation between PGE-MUM and eMayo and between FIT and eMayo. PGE-MUM showed a significant positive correlation with eMayo (*r* = 0.355, *P* < 0.001) (Fig. 2a); a similarly significant positive correlation was shown in FIT (*r* = 0.447, *P* < 0.001) (Fig. 2b). Next, we defined eMayo 0 or 1 as endoscopic remission and eMayo 2 or 3 as endoscopically active and investigated whether PGE-MUM and FIT differed between endoscopic remission and endoscopic activity. PGE-MUM was significantly higher in patients with an eMayo of 2 or 3 than in those with an eMayo of 1 or 2 (*P* = 0.012) (Fig. 2c). FIT was also significantly higher in patients with an eMayo of 2 or 3 than in those with an eMayo of 1 or 2 (*P* < 0.001) (Fig. 2d). The PGE-MUM or FIT values showed a significant correlation with the sum of eMayo (*r* = 0.413, *P* < 0.001 and *r* = 0.390, *P* < 0.001, respectively; Fig. 2e and f). Regarding the correlation to PGE-MUM, the correlation coefficient of the sum of eMayo was slightly larger than the maximum eMayo (*r* = 0.355 vs *r* = 0.413).

**Fig. 3:**
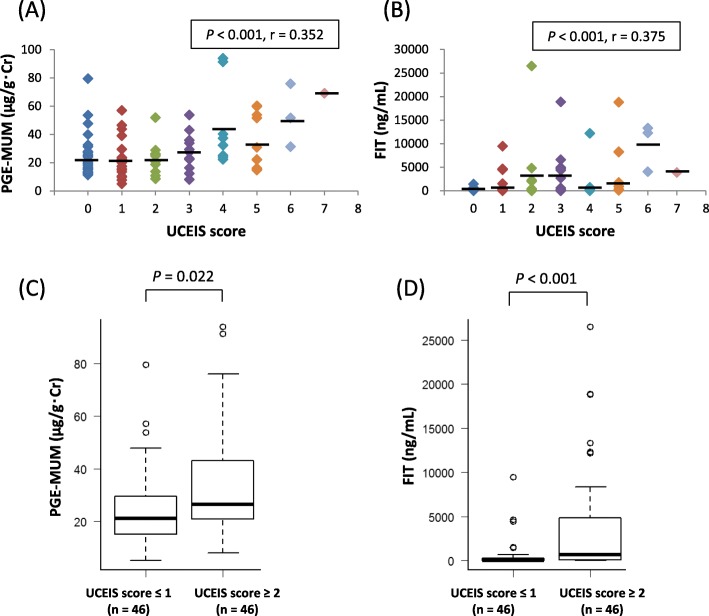
Correlation between urinary/fecal biomarkers and the Ulcerative Colitis Endoscopic Index of Severity (UCEIS) in patients with ulcerative colitis (UC). Scatter plots of (**a**) prostaglandin E-major urinary metabolite (PGE-MUM) vs. UCEIS score, and (**b**) fecal immunochemical occult blood test (FIT) vs. UCEIS score. **c** Comparison of PGE-MUM level between subgroups (UCEIS score ≤ 1 and UCEIS score ≥ 2). **d** Comparison of FIT level between groups with UCEIS score ≤ 1 and UCEIS score ≥ 2

Next, we examined the correlation between PGE-MUM and UCEIS score and that between FIT and UCEIS score. PGE-MUM showed a significant positive correlation with UCEIS score (*r* = 0.352, *P* < 0.001) (Fig. 3a), while FIT and UCEIS score also showed a significant positive correlation (*r* = 0.375, *P* < 0.001) (Fig. 3b). Next, we defined UCEIS score ≤ 1 as endoscopic remission and UCEIS score ≥ 2 as endoscopic activity and investigated whether PGE-MUM and FIT differed between endoscopic remission and endoscopic activity. PGE-MUM level was significantly higher in patients with a UCEIS score ≥ 2 than in those with a UCEIS score ≤ 1 (*P* = 0.022) (Fig. 3c). FIT was also significantly higher in patients with a UCEIS score ≥ 2 than in those with a UCEIS score ≤ 1 (*P* < 0.001) (Fig. 3d).

Next, the accuracies of PGE-MUM and FIT were assessed using receiver-operating characteristic (ROC) curves, defining an eMayo of 0 or 1 as an endoscopic remission of UC (Table 2). The area under the curve (AUC) of PGE-MUM and FIT were 0.644 (95% confidence interval [CI], 0.523–0.76) and 0.862 (95% CI, 0.74–0.912), respectively. The optimal cutoff PGE-MUM value based on eMayo was 30.9 μg/g·Cr. Likewise, the AUC for PGE-MUM and FIT for predicting endoscopic remission by the UCEIS score were 0.636 (95% CI, 0.522–0.765) and 0.789 (95% CI, 0.698–0.879), respectively. The best cutoff for reflecting endoscopic severity by the UCEIS score was 20.8 μg/g·Cr. On the other hand, the AUC of PEG-MUM and FIT in eMayo 0 versus 1 to 3 are 0.614 (95% CI, 0.498–0.729) and 0.790 (95% CI, 0.702–0.878), respectively (data not shown), indicating that eMayo 0, 1 versus 2 and 3 was superior for the accurate evaluation of both biomarkers.

**Table 2:** Test characteristics of two biomarker levels to predict endoscopic remission

	eMayo		UCEIS score	
	PGE-MUM	FIT	PGE-MUM	FIT
Cut-off value	30.9 μg/g∙Cr	742 ng/mL	20.8 μg/g•Cr	31 ng/mL
AUC	0.644	0.826	0.636	0.789
95%CI	0.523–0.765	0.74–0.912	0.522–0.75	0.698–0.879
PPV	55.2%	81.2%	60.3%	70.2%
NPV	73.0%	75.0%	67.6%	82.9%
Sensitivity	48.5%	63.6%	76.1%	87.0%
Specificity	78.0%	88.1%	50.0%	63.0%
Accuracy	67.4%	79.3%	63.0%	75.0%

*eMayo* Mayo endoscopic subscore, *UCEIS* Ulcerative Colitis Endoscopic Index of Severity, *PGE-MUM* Prostaglandin E-Major Urinary Metabolite, *FIT* fecal immunochemical occult blood test, *AUC* area under curve, *CI* confidence interval, *PPV* Positive-predictive value, *NPV* negative-predictive value

### Influence of long-term UC disease duration on urinary/fecal biomarkers

Finally, to investigate whether the UC disease duration affects the detection ability of PGE-MUM or FIT, we divided the patients into two groups (disease duration < 5 years or > 5 years) and performed a subgroup analysis of the endoscopic findings. The differences in characteristics between these two subgroups are shown in Table 3. No significant differences in age as well as eMayo and UCEIS scores were observed between these two subgroups. The disease duration of the group with patients that were affected for < 5 years was significantly higher than in those affected for ≥5 years. CAI and the FIT value of the group with disease duration of < 5 years were also significantly higher than that of patients with a disease duration of ≥5 years.

**Table 3:** Difference of characteristics between the study subgroups

Characteristics	Disease duration		*P* value
	< 5 years, *n* = 48	≥ 5 years, *n* = 44	
Age (year), mean ± SD	46.6 ± 15.2	50.7 ± 17.8	0.232
Male/Female, n (%)	33 / 15 (68.8/31.2)	31/13 (70.5/29.5)	
Disease duration (year), mean ± SD	2.1 ± 1.3	12.7 ± 6.5	< 0.001
Disease extent, n (%)			
Extensive colitis	31 (64.6)	26 (59.1)	
Left sided colitis	8 (16.7)	16 (36.4)	
Proctitis	9 (18.7)	2 (4.5)	
CAI (Lichtiger’s socre) mean ± SD	2.5 ± 2.9	1.3 ± 1.7	0.016
eMayo mean ± SD	1.1 ± 1.0	0.9 ± 1.0	0.250
eMayo 0, n (%)	16 (33.3)	22 (50.0)	
eMayo 1, n (%)	13 (27.1)	8 (18.2)	
eMayo 2, n (%)	16 (33.3)	11 (25.0)	
eMayo 3, n (%)	3 (6.2)	3 (6.8)	
UCEIS score mean ± SD	2.4 ± 1.9	1.8 ± 1.9	0.146
PGE-MUM (μ•g^−1^•Cr^− 1^) mean ± SD	32.3 ± 16.8	25.8 ± 19.5	0.088
FIT (ng/ml), n (%) mean ± SD	3285.8 ± 5953.5	969.6 ± 2118.1	0.017
Medication at study, n (%)			
Oral 5-ASA	25 (52.1)	33 (75)	
Suppository 5-ASA	10 (20.8)	7 (15.9)	
Systemic steroids	9 (18.8)	3 (6.8)	
Azathioprine/Mercaptopurine	22 (45.8)	13 (29.5)	
Tacrolimus	7 (14.6)	3 (6.8)	
Biologics	13 (27.1)	12 (27.3)	

*CAI* clinical activity index, *eMayo* Mayo endoscopic subscore, *UCEIS* Ulcerative Colitis Endoscopic Index of Severity, *PGE-MUM* Prostaglandin E-Major Urinary Metabolite, *FIT* fecal immunochemical occult blood test, *5-ASA* 5-aminosalicylic acid

**Fig. 4:**
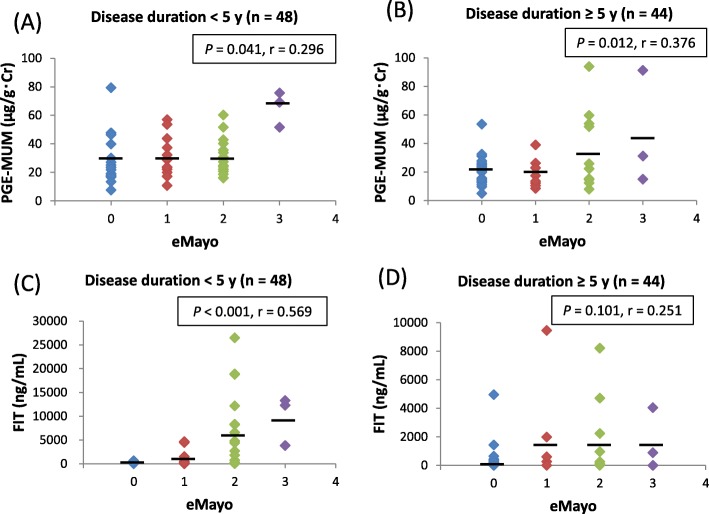
Influence of a long disease duration of ulcerative colitis (UC) on the correlation between urinary/fecal biomarkers and Mayo endoscopic subscore (eMayo). Scatter plots of (**a**) eMayo vs. prostaglandin E-major urinary metabolite (PGE-MUM), and (**b**) eMayo vs. fecal immunochemical occult blood test (FIT) in patients with UC with disease duration of < 5 years. Scatter plots of (**c**) eMayo vs. PGE-MUM and (**d**) eMayo vs. FIT in patients with UC with a disease duration of ≥5 years

**Fig. 5:**
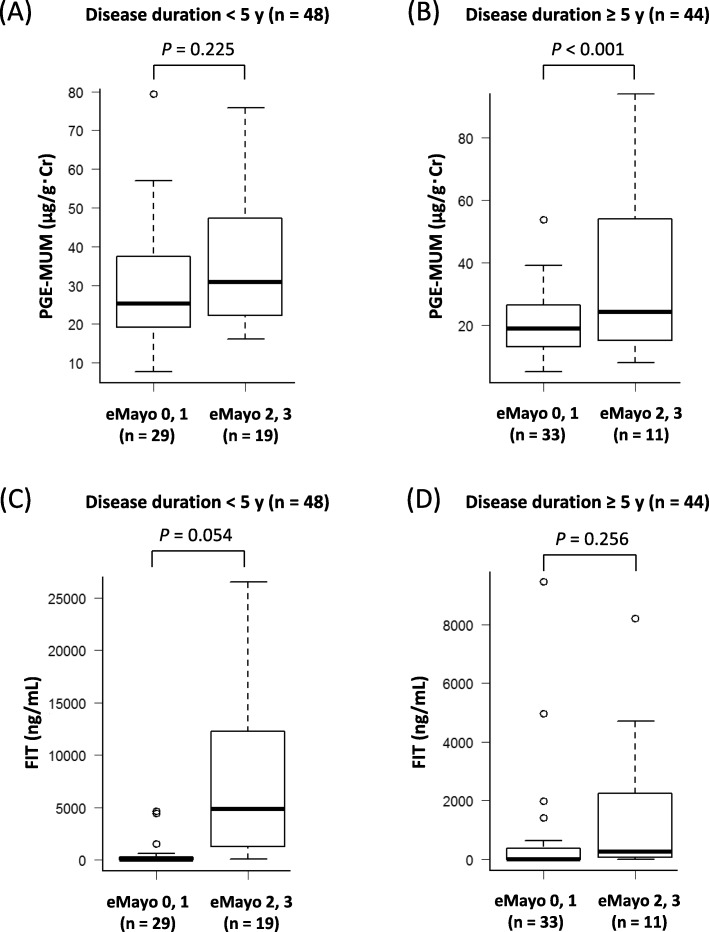
Influence of a long disease duration of ulcerative colitis (UC) on a comparison of urinary/fecal biomarkers between subgroups (Mayo endoscopic subscore [eMayo] ≤ 1 and eMayo ≥2). Comparison of (**a**) prostaglandin E-major urinary metabolite (PGE-MUM) and (**c**) fecal immunochemical occult blood test (FIT) levels between groups with an eMayo ≤1 and eMayo ≥2 in patients with UC with a disease duration of < 5 years. Comparison of PGE-MUM (**b**) and FIT (**d**) levels between the groups with an eMayo ≤1 and eMayo ≥2 in patients with UC with a disease duration > 5 years

We found significant positive correlations between PGE-MUM and eMayo in both groups (< 5 years: *r* = 0.296, *P* = 0.041; ≥ 5 years: *r* = 0.376, *P* = 0.012, respectively) (Figs. 4a and 5b).

Although there was a significant positive correlation between FIT and eMayo in the group with a disease duration of < 5 years (*r* = 0.569, *P* < 0.001) (Fig. 4c), no correlation was seen in the group with a disease duration of ≥5 years (*r* = 0.251, *P* = 0.101) (Fig. 5d). Next, we divided the endoscopic findings into eMayo 0, 1 and eMayo 2, 3 subgroups.

Although the group with a disease duration of < 5 years had a significant difference of PGE-MUM between eMayo of 0 or 1 and eMayo of 2 or 3 (*P* < 0.001) (Fig. 5b), there was no significant difference in the same group (*P* = 0.225) (Fig. 5a). Interestingly, in the group with a disease duration of ≥5 years, FIT values did not differ significantly between the eMayo 0, 1 and eMayo 2, 3 subgroups (Fig. 5d). On the other hand, the PGE-MUM values tended to be higher in the eMayo 2, 3 subgroup than in the eMayo 0, 1 subgroup, but the inergroup differences were not significant (Fig. 5c).

We also examined significant differences between PGE-MUM or FIT and the eMayo 0 and eMayo 1 to 3 groups. In all the cases, the PGE-MUM or FIT value was significantly different between eMayo 0 versus 1 to 3 (*P* = 0.027, *P* < 0.001, respectively; data not shown). No significant difference in PGE-MUM was found between the eMayo 0 and eMayo 1 to 3 groups in both the < 5- and ≥ 5-year groups (*P* = 0.355 and *P* = 0.118, respectively). The value of FIT was significantly different between eMayo 0 and eMayo 1 to 3 in the group with a disease duration of < 5 years (*P* < 0.001). On the other hand, in the group with a disease period of ≥5 years, the PGE-MUM value showed no significant difference between eMayo 0 and eMayo 1 to 3 (*P* = 0.070; data not shown).

## Discussion

The release of prostaglandin E 2 (PGE 2), a major chemical mediator involved in promoting and suppressing inflammation, is reportedly increased in the mucosa of UC patients versus healthy subjects [15]. Since PGE2 is produced at the site of inflammation and metabolized and decomposed upon its release into the blood, taking direct measurements is difficult. However, PGE-MUM, a urinary metabolite of PGE 2, is known to stabilize and reflect systemic PGE 2 production. In fact, although Arai et al. reported that PGE-MUM reflects mucosal inflammation severity in UC, it remains unclear whether PGE-MUM accurately reflects endoscopic severity either immediately after onset or in long-term patients [9]. Our study revealed that PGE-MUM was significantly correlated with endoscopic severity, even in patients with a disease duration ≥5 years. The usefulness of biomarkers such as fecal calprotectin and FIT for IBD was recently reported. Although fecal examinations are non-invasive, feces have the possibility of directly reflecting inflammatory changes of the colonic mucosa and may pick up latent inflammation earlier, leading to the identification of several problems. First, due to heterogeneity, results may vary depending on the site of sample collection.

The value of FC is reportedly wide, even in the same stool sample collected on the same day [16]. Moreover, patients may find it cumbersome to bring stool samples from home to the hospital. On the other hand, urine specimens can be collected at the hospital and have the advantage of being more convenient and non-invasive. These are great benefits for pediatric patients with UC who experience difficulty undergoing frequent invasive endoscopic examinations. Hagiwara et al. reported that PGE-MUM is useful for pediatric patients with UC to evaluate their disease activity [17].

Here we compared the usefulness of PGE-MUM to that of FIT using urine samples. FIT, which is reportedly useful as a biomarker for patients with UC, is less expensive than other fecal biomarkers such as FC, can be performed at many facilities, and boasts immediately available results. Although FC requires 5–10 g of fecal material, FIT can be examined using only smaller samples [18, 19]. FC reflects the volume of inflammatory cells in the intestinal tract, while FIT reflects blood originating from the damaged mucosa. PGE-MUM is similar to FC from the point of view of such an inflammation-derived mechanism. Although current reports show that FIT and FC can efficiently predict mucosal healing of UC, FIT was more sensitive than FC for predicting only eMayo 0 [20]. For the above reasons, we selected FIT rather than FC for comparison with PGE-MUM. Ideal biomarkers are able to detect the recurrence of intestinal mucosal inflammation in an asymptomatic state. In our study, PGE-MUM and FIT were correlated with endoscopic severity and differed significantly between patients with and those without endoscopic activity. Considering its simplicity and minimal invasiveness, PGE-MUM seems clinically useful. From our results, neither PGE-MUM nor FIT was correlated with CRP or ESR, blood biomarkers reflecting mucosal inflammation. However, it is interesting to note that serum Alb, a biomarker representing nutritional status, has a significant negative correlation with both PGE-MUM and FIT. Few reports show correlations between serum Alb and PGE-MUM or serum Alb and FIT, and the mechanism by which serum Alb or nutritional status affects the values of PGE-MUM and FIT is unknown. However, when the serum Alb value deviates significantly from normal or is abnormally low, caution may be required when evaluating PGE-MUM and FIT as biomarkers of UC activity.

The PGE-MUM values at baseline (eMayo 0 score), which are shown in Fig. 2a, were relatively higher than those published in a previous report. In a study by Arai et al., the mean PGE-MUM value was 15.9 μg/g·Cr in the eMayo 0 group, whereas in this study, it was 24.3 μg/g·Cr [9]. Laxative administration has been reported to increase the PGE-MUM value [21]. As almost all the enrolled patients were administered laxatives 1 day before the examination, the mean PGE-MUM value in this study may have been higher than that in a previous study.

PGE-MUM and FIT showed a significant positive correlation with eMayo in the group of patients with a disease duration of < 5 years. Although PGE-MUM and eMayo were significantly correlated in patients with a disease duration of ≥5 years, FIT was not correlated with eMayo in that group. In the group of patients with a disease duration of < 5 years, FIT was significantly higher in the eMayo 2, 3 subgroup than in the eMayo 0, 1, but there was no significant difference between FIT and eMayo in the group with a disease duration of ≥5 years. On the other hand, regarding PGE-MUM, no significant differences were seen between the eMayo 0, 1 and eMayo 2, 3 subgroups in patients with a disease duration of < 5 years, but PGE-MUM tended to be higher in eMayo 2, 3 than in eMayo 0, 1 in patients with a disease duration ≥5 years (*P* = 0.054). Patients with a long disease duration often experience repeated relapse and remission, and there are many sites of scarring and rough-surfaced mucosa in their intestinal tract. In these patients, the degree of bleeding is small although endoscopic abnormalities are recognized. Since FIT reflects the amount of bleeding itself, it may be difficult to reflect endoscopic abnormalities associated with inflammation in long-term diseased patients. On the other hand, since PGE-MUM reflects intestinal tract inflammation, it is likely able to reflect endoscopic abnormalities associated with inflammation in UC patients with long disease duration. Our results show that PGE-MUM may be a more useful biomarker for predicting endoscopic severity when disease duration is prolonged; however, the accumulation of cases is necessary to confirm these results.

There are some limitations to this study. First, it was performed in a single center and included a small number of patients. Also, in this study, it was not proven whether PGE-MUM can predict UC recurrence. To clarify that point, there is a need for periodic measurements of PGE-MUM to be performed in the same patient and prospective follow-up be performed of patients with relapse. In recent years, treatments aimed at histological healing more advanced than mucosal healing have also been required, and reports of the comparison between histological healing and biomarkers have been found [22–25]. Further studies are needed to determine if PGE-MUM could be a biomarker to predict histologic healing.

## Conclusion

PGE-MUM is a biomarker capable of predicting endoscopic activity as well as FIT and could be more sensitive than FIT to represent the endoscopic UC activity in long-term affected patients. Further studies are expected to effectively utilize PGE-MUM measurements to maintain mucosal healing in UC patients.

## Data Availability

Not applicable.

## Abbreviations

UC: Ulcerative colitis
CRP: C-reactive protein
FC: Fecal calprotectin
FIT: Fecal immunochemical occult blood test
PGE-MUM: Prostaglandin E-major urinary metabolite
CAI: Clinical activity index
eMayo: Mayo endoscopic subscore
UCEIS: Ulcerative Colitis Endoscopic Index of Severity
ESR: Erythrocyte sedimentation rate
Alb: Albumin
ROC: Receiver-operating characteristic
AUC: Area under the curve
CI: Confidence interval
